# H_2_O_2_ Production at Low Overpotentials for Electroenzymatic Halogenation Reactions

**DOI:** 10.1002/cssc.201902326

**Published:** 2019-10-17

**Authors:** Sebastian Bormann, Morten M. C. H. van Schie, Tiago Pedroso De Almeida, Wuyuan Zhang, Markus Stöckl, Roland Ulber, Frank Hollmann, Dirk Holtmann

**Affiliations:** ^1^ Industrial Biotechnology DECHEMA Research Institute Theodor-Heuss-Allee 25 60486 Frankfurt am Main Germany; ^2^ Department of Biotechnology, Biocatalysis Group Technical University Delft Van der Maasweg 9 2629HZ Delft The Netherlands; ^3^ Electrochemistry DECHEMA Research Institute Theodor-Heuss-Allee 25 60486 Frankfurt am Main Germany; ^4^ Bioprocess Engineering University of Kaiserslautern Gottlieb-Daimler-Str. 49 67663 Kaiserslautern Germany

**Keywords:** biocatalysis, carbon nanotubes, electrochemistry, enzymes, hydrogen peroxide

## Abstract

Various enzymes utilize hydrogen peroxide as an oxidant. Such “peroxizymes” are potentially very attractive catalysts for a broad range of oxidation reactions. Most peroxizymes, however, are inactivated by an excess of H_2_O_2_. The electrochemical reduction of oxygen can be used as an in situ generation method for hydrogen peroxide to drive the peroxizymes at high operational stabilities. Using conventional electrode materials, however, also necessitates significant overpotentials, thereby reducing the energy efficiency of these systems. This study concerns a method to coat a gas‐diffusion electrode with oxidized carbon nanotubes (oCNTs), thereby greatly reducing the overpotential needed to perform an electroenzymatic halogenation reaction. In comparison to the unmodified electrode, with the oCNTs‐modified electrode the overpotential can be reduced by approximately 100 mV at comparable product formation rates.

## Graphical abstract

**Low and behold**: Coating of a gas‐diffusion electrode with oxidized carbon nanotubes (oCNTs) is found to greatly reduce the overpotential needed to perform an electroenzymatic halogenation process. In comparison to the unmodified electrode, with the oCNTs‐modified electrode the overpotential can be reduced by around 100 mV at comparable product formation rates.

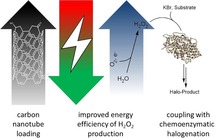

Hydrogen peroxide is increasingly considered as an oxidant for biocatalytic reactions. The promise of H_2_O_2_‐driven biocatalysis is that the high oxidation power of H_2_O_2_ and its ecological properties can be combined with the high selectivity and further advantages of the enzymatic oxidation reactions.^1^ This point motivates many researchers to investigate H_2_O_2_‐driven biocatalysis for reactions such hydroxylations, epoxidations or halogenations.^1^, ^2^ A major issue for the technical application of these “peroxizymes” is H_2_O_2_‐mediated inactivation of the enzymes. Given that H_2_O_2_ is a strong oxidant, it is not surprising that, in particular, labile amino acids can be oxidized by H_2_O_2_ or reactive oxygen species interact with the protein.^3^ Therefore, a major challenge en route to practical reaction systems is to control the H_2_O_2_ concentration at levels that enable efficient catalytic turnover of the enzyme while simultaneously minimizing the undesired inactivation reaction. Several H_2_O_2_ dosing systems have been proposed to enable high productivities combined with high operational stabilities of the hydrogen peroxide‐dependent enzymes, for example, enzymatic,^4^ photochemical^5^ or electrochemical H_2_O_2_ production systems.^6^ In recent years the electrochemical reduction of oxygen to H_2_O_2_ at gas‐diffusion electrodes (GDEs) has gained more and more attention.^7^ GDEs are typically being used in alkaline and proton exchange membrane fuel cells.

The GDEs are installed in an electrochemical cell in a way that one side is facing the liquid electrolyte and the other side is facing the gas phase (e.g., air or pure oxygen). GDEs have a porous structure into which the electrolyte can float from one side and the desired gas can diffuse into the electrode from the other side. This setting leads to a huge 3‐phase boundary between the solid catalyst, liquid electrolyte, and gas phase. Scheme 1 shows the principle of the GDE for the reduction of molecular oxygen to hydrogen peroxide. The GDEs were successfully combined with different H_2_O_2_‐dependent enzymes, such as the unspecific peroxygenase from *Agrocybe aegerita* 8 and a chloroperoxidase from *Caldariomyces fumago*.^9^ To date, process engineering parameters, such as electrochemical potential, buffer composition, and flow rate, have been optimized. However, a key parameter that has been neglected in the investigations is the applied electrocatalyst. To date, most applied catalyst have consisted of carbon and these have not been investigated or optimized. Very recently, it was shown that the oxidation of the surface of a carbon catalyst to ‐C−O−C‐ groups inside the structure and ‐COOH groups at the edges leads to a significant decrease in the overpotentials required for O_2_ reduction.^10^ Therefore, the application of oxidized carbon nanotubes (oCNT) resulted in more energy‐efficient H_2_O_2_ production. These results motivated us to investigate the novel electrocatalyst in combination with a H_2_O_2_‐dependent enzyme. This approach combines the advantages of GDEs (high atom efficiency in H_2_O_2_ synthesis, overcoming solubility issues of O_2_) and enzyme catalysis (high selectivity and specificity) with the reduced energy demand of the novel electrocatalyst. The standard redox potential for oxygen reduction to hydrogen peroxide in acidic aqueous solution is 0.7 V vs. the standard hydrogen electrode (0.5 V vs. saturated Ag/AgCl). In an electrochemical cell, the existence of so‐called overpotentials implies the electrochemical cell requires more energy than thermodynamically expected from the standard potential to drive a reaction. Therefore, the optimization of overpotentials such as the electrode overpotentials must be addressed.

**Scheme 1:**
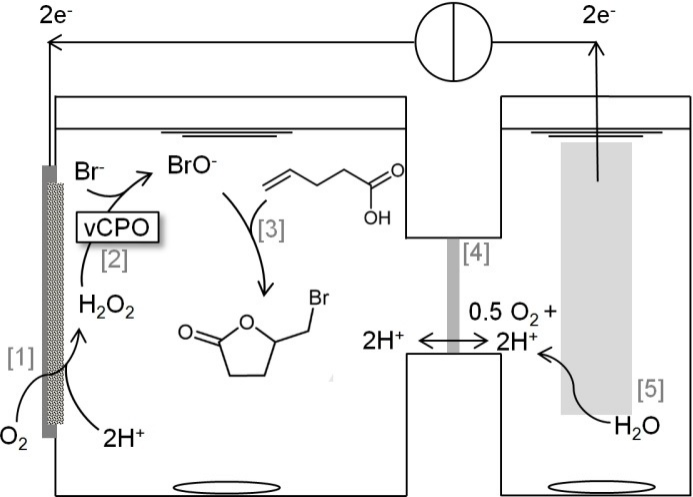
Schematic overview of the reaction system for electrobiocatalytic 5‐(bromomethyl)dihydrofuran‐2(3*H*)‐one (bromolactone) synthesis. H_2_O_2_ is produced at a gas‐diffusion electrode (GDE) [1] which consists of oxidized carbon nanotubes (oCNTs) immobilized on carbon paper by drop coating. Oxygen from ambient air can diffuse through the GDE. Increasing oCNT loadings reduces the overpotential required for H_2_O_2_ production. Hypobromite is generated by *Ci*VCPO [2] and reacts with 4‐pentenoic acid to form bromolactone [3]. A proton exchange membrane [4] separates anode and cathode chambers. Protons and electrons required for H_2_O_2_ synthesis are replenished by water oxidation at a platinum anode [5]. The reference electrode is omitted for clarity.

As biocatalyst, we chose the vanadium chloroperoxidase from *Curvularia inaequalis* (*Ci*VCPO). This vanadium‐dependent chloroperoxidase is able to efficiently produce hypohalides from hydrogen peroxide and a halide. These reactive compounds are then able participate in various halogenation reactions.^11^ For reasons of reaction stability, prevention of futile H_2_O_2_ dismutation, and enzyme activity, it is preferable to keep the concentration of the peroxide minimal in the reaction solution.

To show the effect of different amounts of immobilized oCNTs on the gas‐diffusion electrodes, linear sweep experiment were performed (Figure 1). Here the cell current was measured as a function of the applied potential between the working and the counter electrode. The higher the amount of oCNTs deposited on the electrodes, the lower the potential required to reduce the dissolved oxygen in the electrolyte. By adding 1 mg cm^−2^ oCNTs, the electrode overpotential can be reduced by approximately 0.1 V (calculated from the intersection of the linear range of the current curve and the *y* axis). Thus, the promising values in 0.1 m KOH^10^ were transferred to an enzyme‐compatible electrolyte.

**Figure 1:**
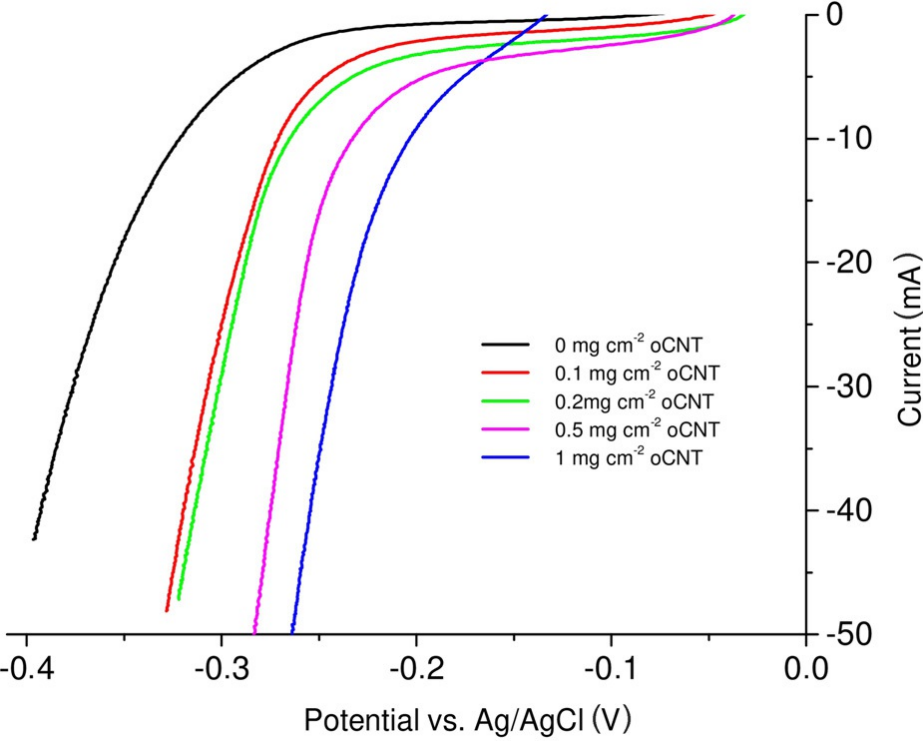
Linear sweep experiments of electrodes modified with different amounts of oCNTs in 100 mm sodium citrate (pH 5) with 100 mm KBr. Potentials are iR‐compensated.

To evaluate the hydrogen peroxide production rates at electrode modified with different amounts of oCNTs the electrodes were compared in an electrochemical set‐up. Different potentials were applied and the resulting H_2_O_2_ production rates were measured (Figure 2). The addition of oCNTs not only has a positive influence on the overpotential in the electrochemical measurements (Figure 1), but also leads to improved H_2_O_2_ production. For example, the addition of 0.1 mg cm^−2^ oCNTs leads to a 2.3‐fold higher production rate of H_2_O_2_ at a potential of −0.35 vs. Ag/AgCl. An increase in the oCNTs loading to 1 mg cm^−2^ leads to a 4.6‐fold increase in the production rate at the chosen potential. The application of oCNTs thus leads to an improved H_2_O_2_ production rate at different potentials and to better utilization of the applied electrical energy. In all experiments, the current efficiencies were around 80 % (data not shown).

**Figure 2:**
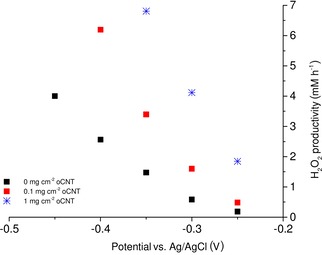
Hydrogen peroxide production at oCNT‐modified electrodes at different potentials. Production rates were determined by linear regression, the regression coefficient was at least *R* ^2^>0.99 (for the corresponding data, see the Supporting Information, Table S1). Potentials are iR‐compensated.

In the next step of our investigations, electrochemical production of H_2_O_2_ was combined with enzymatic conversion of 4‐pentenoic acid to bromolactone (Scheme 1). Figure 3 compares the product formation at an unmodified electrode and that at an electrode with 1 mg oCNTs cm^−2^ at −250 and −350 mV vs. Ag/AgCl. The highest production rate can be measured at the modified electrode at the more negative potential. Bromolactone was produced at a rate of approximately 4.5 mm h^−1^. The productivity was more than 8 times higher at the modified electrode than at the plain electrode. This is mainly due to the improved H_2_O_2_ production (see Figure 2). An improvement in the product formation rate can also be observed at −250 mV vs. Ag/AgCl. Here, the modified electrode produces roughly 4 times more product in the first 5 h than the unmodified electrode. Comparing the H_2_O_2_ generation rates (Figure 2) with the actual product accumulation rates reveals that about 50 % of the electrochemically generated H_2_O_2_ is used productively. This rather poor efficiency can most likely be attributed to the undesired, spontaneous reaction between H_2_O_2_ and hypobromite.^12^ Further adjustments of the reaction parameters (particularly of the concentrations of *Ci*VCPO and Br^−^) will reduce this futile reaction. As is shown in Figure 3, the catalyst‐doped electrode performs roughly as well at −250 mV vs. Ag/AgCl as the untreated GDE at −350 mV vs. Ag/AgCl. Usually, noble metals are used to decrease the overpotential of different electrode reactions; here we applied abundant carbon‐based materials. This can be regarded as another ecological and economic advantage.

**Figure 3:**
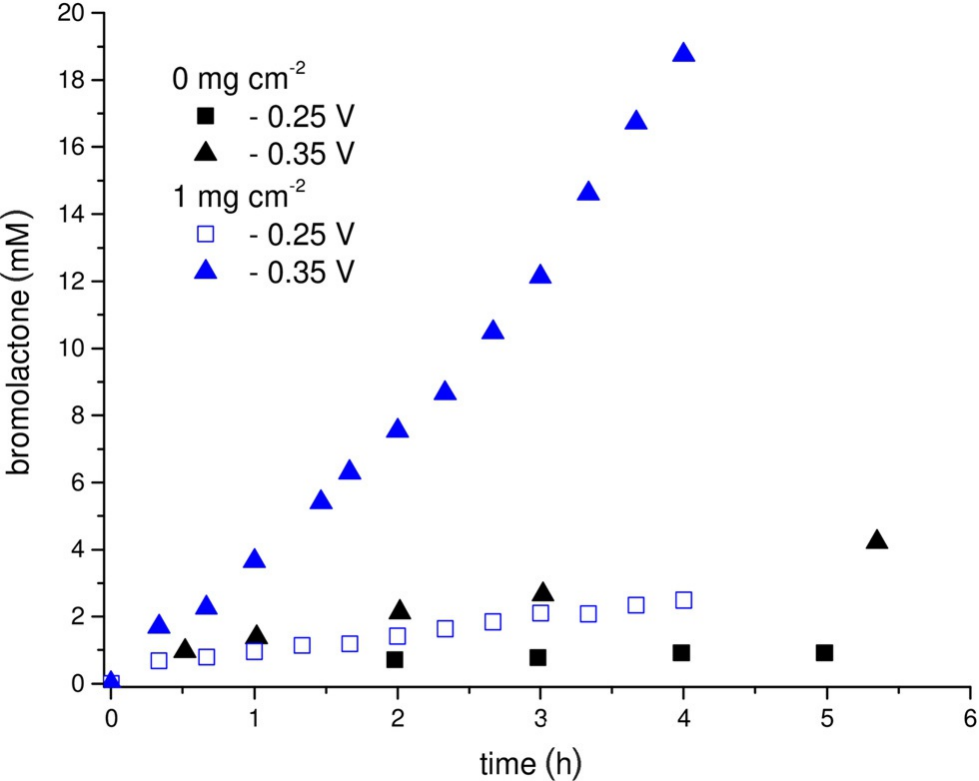
Electroenzymatic product formation at an unmodified electrode and an oCNT‐coated electrode at −0.25 and −0.35 V vs. Ag/AgCl. The *Ci*VCPO concentration was 25 nm (110.5 U_MCD_ L^−1^) for experiments at −0.25 V and 100 nm (≙442 U_MCD_ L^−1^) for experiments at −0.35 V; *n*=1.

As these results demonstrate, doping of the catalyst allows reduction of the overpotential that has to be applied to this system by about 100 mV while essentially maintaining the same productivity—thus directly increasing the energy efficiency of this electroenzymatic catalysis.

## Experimental Section

All chemicals were purchased for Sigma–Aldrich at the highest purity available. The oxidized carbon nanotubes (*o*‐CNT) were a gift from the group of Yi Cui (Stanford University, Stanford, CA, USA). The Sigracet GDL 38 BC carbon paper was a gift from SGL Carbon (Germany).

### Synthesis of 5‐(bromomethyl)dihydrofuran‐2(3*H*)‐one (bromolactone)

The synthesis of the bromolactone as an authentic standard was performed at room temperature for 24 h with stirring. A 100 mm citrate buffer (pH 5, final volume of 50 mL) contained 160 mm KBr, 10 mmol 4‐pentenoic acid, 100 nm *Ci*VCPO and 100 mm of H_2_O_2_. At the end of the reaction, the mixture was extracted by dichloromethane (3×, 100 mL) and dried over anhydrous Na_2_SO_4_. The combined organic layers were concentrated under reduced pressure. The bromolactone compound was isolated by using flash column chromatography on silica gel (EtOAc/hexanes, 1:2) and analyzed by ^1^H NMR spectroscopy to give 1.4 g of isolated product (80 % yield).

### Enzyme preparation

100 mL pre‐cultures of LB (tryptone 10 g L^−1^, yeast extract 5 g L^−1^, NaCl 10 g L^−1^) medium containing 50 μg mL^−1^ ampicillin were inoculated with *E. coli* TOP10 pBADgIIIB VCPO and incubated overnight at 37 °C and 180 rpm. Overexpression was carried out in 5 L flasks with 1 L of TB (tryptone 12 g L^−1^, yeast extract 24 g L^−1^, glycerol 5 g L^−1^, in 89 mm KPi buffer pH 7.5) medium supplemented with 50 μg mL^−1^ of ampicillin and grown at 37 °C and 180 rpm. At an OD_600_ of 0.9 A.U., 0.02 wt % of l‐arabinose was added. After induction, cultures were incubated for additional 24 h at 25 °C and 180 rpm. The bacterial pellets obtained after centrifugation were re‐suspended in 50 mm Tris/H_2_SO_4_ buffer (pH 8.1). 0.1 mm phenylmethylsulfonyl fluoride (100 mm stock in isopropanol) was added to the re‐suspended cells, which were ruptured by sonication on ice (output 4, cycle 40 %). The samples were then centrifuged (10 000 rpm for 20 minutes) and the supernatant was incubated at 70 °C for 1.5 h. After centrifugation (10 000 rpm for 10 minutes), the absence of catalase activity was determined by adding the enzyme to a solution of 0.1 % Triton and 3 % H_2_O_2_ in a KPi buffer (50 mm, pH 7.0).^13^ The clarified protein solution was further purified with a Q Sepharose FF column. After washing with 2 column volumes of 50 mm Tris/H_2_SO_4_, pH 8.1 and 2 column volumes of 0.1 m NaCl in 50 mm Tris/H_2_SO_4_, pH 8.1, the enzyme was loaded at 7.5 mL min^−1^ and thereafter eluted with 0.6 m NaCl in 50 mm Tris/H_2_SO_4_, pH 8.1. Fractions containing *Ci*VCPO (determined by the MCD activity assay) were pooled, concentrated (Amicon 10 kDa cut‐off membrane), and desalted by using HiTrap desalting or PD10 columns (GE Healthcare) and 50 mm Tris/H_2_SO_4_, pH 8.1 containing 100 μm orthovanadate.

*Ci*VCPO activity was quantified by the monochlorodimedone (MCD) assay. The enzyme solution was added to a reaction mixture containing MCD (50 μm), KBr (5 mm), and orthovanadate (100 μm) in a 100 mm citrate buffer at pH 5.0. After addition of H_2_O_2_ (5 mm), the enzyme activity could be determined by following the decrease in absorbance at 290 nm. After purification, the *Ci*VCPO solution used in this study had a purity of 64 %, as determined by gel densitometry (see the Supporting Information, Figure S1), with a specific MCD‐activity of 65.5±6.1 U mg^−1^.

### Electrode preparation

A solution of oxidized carbon nanotubes (*o*‐CNTs; 2 mg mL^−1^ or 5 mg mL^−1^) was suspended in ethanol containing 1 % *w*/*v* Nafion 117 (Sigma–Aldrich, Germany) and sonicated for 60 min. This suspension was evenly pipetted onto 5 cm×5 cm sheets of Sigracet GDL 38 BC. Electrodes were left out to dry overnight and used without further conditioning.

### (Bio)electrochemical setup

Experiments were carried out in a H‐cell^14^ divided by a proton exchange membrane (Nafion 117, Sigma–Aldrich, St. Louis, USA). The anode consisted of a 4 cm×2 cm platinum sheet contacted by a glass‐coated platinum wire. The anode chamber was filled with 100 mL of a 100 mm Na‐Citrate buffer of pH 5. The cathode was prepared as described above and used as a gas‐diffusion electrode (GDE) by mounting it to the circular opening at the side of the H‐cell (2.4 cm diameter). The coated side of the paper was in contact with the aqueous phase. A Ni‐mesh was added to the air side of the GDE to ensure proper contact. The cathode chamber was filled with 100 mL 100 mm Na‐Citrate buffer pH 5 containing 100 mm KBr. An Ag/AgCl electrode in a Luggin capillary filled with 0.5 m Na_2_SO_4_ was used as a reference electrode. The tip of the Luggin capillary was placed at a distance of about 4 mm from the cathode. All experiments were carried out by using a GAMRY Reference 600 potentiostat/galvanostat. Prior to all experiments, the internal resistance was determined by impedance spectroscopy (about 17 Ω) by using the manufacturer provided program “Get Ru”. Linear sweep/cyclic voltammetry measurements were carried out without iR correction and were iR corrected after the experiments. Chronoamperometric experiments were carried out by using positive‐feedback iR compensation by applying 90 % of the internal resistance that had been determined prior to starting the experiments. Bioelectrochemical experiments were carried out in the same setup. For these experiments, 50 mm 4‐pentenoic acid and either 25 nm *Ci*VCPO (1.69 mg L^−1^, 110.5 U_MCD_ L^−1^ ) for experiments carried out at −250 mV vs. Ag/AgCl or 100 nm *Ci*VCPO (6.75 mg L^−1^, 442 U_MCD_ L^−1^) for experiments carried out at 350 mV vs. Ag/AgCl, were added to the cathode chamber containing 100 mm sodium citrate buffer (100 mL, pH 5) with 100 mm KBr. Experiments were started by applying a constant voltage using positive feedback iR compensation as described above.

### Electrochemical H_2_O_2_ measurement

Hydrogen peroxide concentrations were determined electrochemically by using a Select 2700 Biochemistry Analyzer (Yellow Springs Instruments, OH, USA) equipped with blank membranes. The calibration solution consisted of 30 mg L^−1^ H_2_O_2_ in a 20 mm Na‐citrate buffer. Sample size taken was 25 μL and the measurement time was 30 s. A typical calibration sample yielded a 10 nA signal with a baseline current below 2 nA.

### Gas chromatography

Aqueous samples (500 μL) were acidified by addition of 6 m HCl (50 μL) and extracted with ethyl acetate (500 μL) containing 10 mm acetophenone as an internal standard. Concentrations of 4‐pentenoic acid and the bromolactone were determined by gas chromatography coupled with flame ionization detection (FID; GC‐17A, Shimadzu, Japan). The compounds were separated on a DB‐WAXetr column (30 m×0.25 mm×0.25 μm; Agilent, CA, USA) with a split ratio of 1:20, at a linear velocity of 31.5 cm s^−1^ with helium as a carrier gas by using the following temperature profile: 130 °C to 190 °C at 7 °C min^−1^; to 230 °C at 15 °C min^−1^; hold 3 min. The resulting retention times were 4.0 min for acetophenone, 5.1 min for 4‐pentenoic acid, and 10.4 min for bromolactone.

## Conflict of interest

*The authors declare no conflict of interest*.

## Supporting information

As a service to our authors and readers, this journal provides supporting information supplied by the authors. Such materials are peer reviewed and may be re‐organized for online delivery, but are not copy‐edited or typeset. Technical support issues arising from supporting information (other than missing files) should be addressed to the authors.

Supplementary
