# Supplementary material for: H2O2 Production at Low Overpotentials for Electroenzymatic Halogenation Reactions
Source: ChemSusChem. 2019 Oct 17;12(21):4759–63. doi: 10.1002/cssc.201902326 (PMC6899481; doi:10.1002/cssc.201902326)
Supplement: Supplementary file 1 — Supplementary [file CSSC-12-4759-s001.pdf]

CHEMISTRY & SUSTAINABILITY
CHEMSUSCHEM
ENERGY & MATERIALS

Supporting Information

H_2O_2 Production at Low Overpotentials for Electroenzymatic Halogenation Reactions

Sebastian Bormann+,[a] Morten M. C. H. van Schie+,[b] Tiago Pedroso De Almeida,[b] Wuyuan Zhang,[b] Markus Stöckl,[c] Roland Ulber,[d] Frank Hollmann,*[b] and Dirk Holtmann*[a]

cssc_201902326_sm_miscellaneous_information.pdf

H_2O_2 production at low over-potentials for electro-enzymatic halogenation reactions

Supplementary Information

Sebastian Bormann[a, #], Morten M.C.H. van Schie[b, #], Tiago Pedroso De Almeida[b], Wuyuan Zhang[b], Markus Stöckl[c], Roland Ulber[d], Frank Hollmann*[b], and Dirk Holtmann*[a]

[a] Sebastian Bormann, Dr. Dirk Holtmann
Industrial Biotechnology
DECHEMA Research Institute
Theodor-Heuss-Allee 25, 60486 Frankfurt am Main, Germany
E-mail: holtmann@dechema.de

[b] Morten M.C.H. van Schie, Tiago Pedroso De Almeida, Prof. Dr. Frank Hollmann
Department of Biotechnology, Biocatalysis Group
Technical University Delft
Van der Maasweg 9, 2629HZ Delft, The Netherlands
E-mail: f.hollmann@tudelft.nl

[c] Dr. Markus Stöckl
Electrochemistry
DECHEMA Research Institute
Theodor-Heuss-Allee 25, 60486 Frankfurt am Main, Germany

[d] Prof. Dr. Roland Ulber
Bioprocess Engineering
University of Kaiserslautern
Gottlieb-Daimler-Str. 49, 67663 Kaiserslautern, Germany

# Both authors contributed equally to this study.

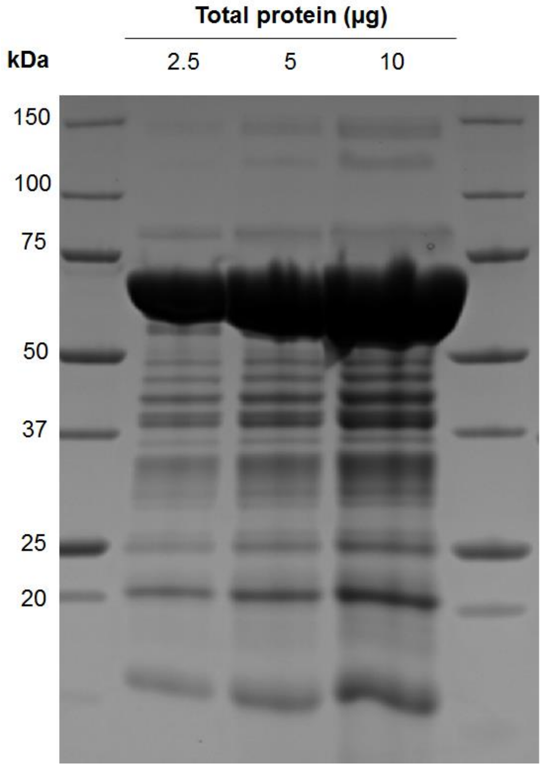

SI Figure 1: SDS-PAGE of *Ci*VCPO, after 90 min of heat purification and anion exchange chromatography, used for gel densitometric purity estimation.

SI Table 1: Measurement of H_2O_2 production rates at various constant potentials. Prior to starting the experiments, the internal resistance was determined for every electrode. A constant, IR-corrected voltage was applied to the electrode and the H_2O_2 concentration was determined in duplicate at regular intervals. H_2O_2 production rates were determined by linear regression, the coefficient of determination is given.

**oCNT loading (µg cm^{-2})**	**Voltage, IR corrected, vs Ag/AgCl (V)**	**time (min)**	**H2O2 (mM)**	**P_{H2O2} (mM h^{-1})**	**R^2 (-)**
0	-0.25	1.53	0.30588235		
		1.53	0.29411765		
		3.12	0.31176471		
		3.12	0.3		
		4.65	0.31470588		
		4.65	0.30294118		
		6.23	0.31764706		
		6.23	0.30882353		
		7.77	0.32647059		
		7.77	0.31470588	0.18682727	0.591072
0	-0.3	9.37	0.35882353		
		9.37	0.35		
		10.88	0.36176471		
		10.88	0.35294118		
		13.78	0.38529412		
		13.78	0.37941176		
		15.63	0.40588235		
		15.63	0.40294118		
		17.17	0.42058824		
		17.17	0.41470588		
		18.70	0.43823529		
		18.70	0.42941176		
		20.28	0.45588235		
		20.28	0.45		
		21.82	0.46764706		
		21.82	0.46176471		
		23.35	0.48529412		
		23.35	0.47647059		
		24.93	0.50294118		
		24.93	0.49411765		
		28.05	0.53235294		
		28.05	0.52647059	0.58434234	0.99083558
0	-0.35	10.03	0.59705882		
		10.03	0.59117647		
		11.75	0.63235294		
		11.75	0.62647059		

		13.47	0.67941176		
		13.47	0.67352941		
		15.22	0.71470588		
		15.22	0.71176471		
		16.93	0.76470588		
		16.93	0.75882353		
		18.65	0.80588235		
		18.65	0.8	1.46850765	0.99692731
0	-0.4	8.38	0.99411765		
		8.38	0.99705882		
		10.20	1.08235294		
		10.20	1.07941176		
		11.92	1.15882353		
		11.92	1.15294118		
		13.63	1.23235294		
		13.63	1.22941176		
		15.40	1.29705882		
		15.40	1.29117647		
		17.12	1.38529412		
		17.12	1.37647059		
		18.83	1.44705882		
		18.83	1.43823529	2.56386373	0.99834145
0	-0.45	15.72	1.78235294		
		15.72	1.79117647		
		17.63	1.9		
		17.63	1.91176471		
		19.48	2.03823529		
		19.48	2.05294118		
		21.38	2.15882353		
		21.38	2.17647059		
		23.28	2.26764706		
		23.28	2.29117647		
		25.17	2.38823529		
		25.17	2.41764706		
		27.08	2.53529412		
		27.08	2.57352941	3.99969593	0.99675057
100	-0.25	11.35	0.45294118		
		13.07	0.47058824		
		13.07	0.46470588		
		14.78	0.48823529		
		14.78	0.48235294		
		16.50	0.50294118		
		16.50	0.49705882		
		18.22	0.51764706		

		18.22	0.51176471		
		19.93	0.53235294		
		19.93	0.52352941		
		21.65	0.54411765		
		21.65	0.53823529		
		23.37	0.55882353		
		23.37	0.55294118		
		25.08	0.57058824		
		25.08	0.56764706		
		26.80	0.58529412		
		26.80	0.57941176		
		28.52	0.59117647	0.49101354	0.9927166
100	-0.35	5.10	0.65588235		
		5.10	0.64705882		
		6.83	0.70588235		
		6.83	0.69705882		
		8.53	0.75294118		
		8.53	0.74411765		
		10.25	0.8		
		10.25	0.79117647		
		11.97	0.84411765		
		11.97	0.83529412		
		13.73	0.88823529		
		13.73	0.87647059		
		15.45	0.94117647		
		15.45	0.92941176		
		17.15	0.98529412		
		17.15	0.97352941		
		18.87	1.02352941		
		18.87	1.00882353		
		20.57	1.07647059		
		20.57	1.06176471	1.60161116	0.99764157
100	-0.35	18.55	1.19705882		
		20.30	1.28823529		
		20.30	1.28823529		
		22.02	1.39705882		
		22.02	1.39705882		
		23.73	1.49117647		
		23.73	1.48823529		
		25.45	1.58823529		
		25.45	1.58529412		
		27.17	1.69117647		
		27.17	1.68823529		
		28.88	1.78529412		
		28.88	1.78235294		

		30.60	1.86470588		
		30.60	1.86470588	3.3751831	0.99913985
100	-0.45	11.50	2.71764706		
		11.50	2.73235294		
		13.37	2.89117647		
		13.37	2.91470588		
		15.30	3.11764706		
		15.30	3.14705882		
		17.18	3.29411765		
		17.18	3.32352941		
		19.07	3.47058824		
		19.07	3.52941176	6.19376547	0.99488963
1000	-0.25	3.43	0.48529412		
		5.15	0.53529412		
		5.15	0.52647059		
		6.87	0.59117647		
		6.87	0.58235294		
		8.57	0.64411765		
		8.57	0.63235294		
		10.28	0.69705882		
		10.28	0.68529412		
		12.00	0.75294118		
		12.00	0.74117647		
		13.75	0.80882353		
		13.75	0.79705882		
		15.47	0.85588235		
		15.47	0.84117647		
		17.18	0.91176471		
		17.18	0.89411765		
		18.90	0.96764706		
		18.90	0.94705882		
		20.62	1.02647059		
		20.62	1.00588235		
		22.32	1.07647059		
		22.32	1.05294118	1.85728487	0.99795054
1000	-0.3	13.73	1.10882353		
		15.43	1.22647059		
		15.43	1.20882353		
		17.15	1.34117647		
		17.15	1.32058824		
		18.90	1.45		
		18.90	1.42647059		
		20.62	1.56176471		
		20.62	1.53529412		

		22.33	1.67941176		
		22.33	1.64705882		
		25.52	1.90588235		
		25.52	1.92058824		
		27.33	2.03235294		
		27.33	2.04117647	4.11791456	0.99685767
1000	-0.35	10.32	2.14117647		
		10.32	2.16470588		
		12.03	2.32352941		
		12.03	2.35		
		13.77	2.5		
		13.77	2.53529412		
		18.25	3.02941176		
		18.25	3.02941176		
		20.12	3.23529412		
		20.12	3.26470588		
		22.05	3.47058824		
		22.05	3.5	6.80870956	0.99879538
